# Earthworm effects on native grassland root system dynamics under natural and increased rainfall

**DOI:** 10.3389/fpls.2014.00152

**Published:** 2014-04-22

**Authors:** John A. Arnone, Johann G. Zaller

**Affiliations:** ^1^Institute of Botany, University of BaselBasel, Switzerland; ^2^Division of Earth and Ecosystem Sciences, Desert Research InstituteReno, NV, USA; ^3^Department of Integrative Biology and Biodiversity Research, Institute of Zoology, University of Natural Resources and Life Sciences ViennaVienna, Austria

**Keywords:** belowground–aboveground interactions, grassland ecology, plant–animal interactions, root ecology, soil ecology, root growth, plant growth (biomass) allocation

## Abstract

Earthworms (EWs) can modify soil structure and nutrient availability, and hence alter conditions for plant growth through their burrowing and casting activities. However, few studies have specifically quantified EW effects by experimentally manipulating earthworm densities (EWDs). In an earlier field study in native grassland ecosystems exposed to ambient and experimentally elevated rainfall (+280 mm year^-1^, projected under some climate change scenarios), we found no effects of EWDs (37, 114, 169 EW m^-2^) and corresponding EW activity on aboveground net primary productivity (ANPP), even though soil nutrient availability likely increased with increasing EWDs. The lack of effects of EWDs on ANPP suggested that EWs may have adversely affected root systems in that study in some way. The objective of the present study was to quantify responses of root length density (RLD), using data collected from the same grassland plots during the earlier study. RLDs were highest in plots with low EWDs and decreased in plots with higher EWDs. Elevated rainfall primarily increased RLDs in the low EWD treatment (by almost +40%). Reductions in RLDs resulting from increased EWDs did not affect ANPP. Our results indicate that elevating EWDs above ambient levels may limit root growth through large increases in soil bioturbation, but concurrent increases in cast production and nutrient availability may compensate for the suppression of root nutrient absorbing surface area leaving ANPP unchanged, but with shifts in growth (biomass) allocation toward shoots. Similarly, reductions in EWDs appeared to promote higher RLDs that increased soil nutrient foraging in soil with lower amounts of nutrients because of reduced casting activity. Amplified responses observed when rainfall during the growing season was increased suggest that EWDs may mainly affect RLDs and above- vs. belowground growth (biomass) allocation under climate changes that include more frequent wetter-than-average growing seasons.

## INTRODUCTION

Ever since the late 1800s, with the pioneering work of Hensen (1877) and Darwin (1881), earthworms (EWs) have been known for their large “engineering” effects (Jones et al., 1994) on the chemistry and physical structure of soils. These effects include stimulation of litter and soil organic matter decomposition and soil nutrient mineralization that can enhance soil nutrient availability (e.g., Lee, 1995; Edwards and Bohlen, 1996) and plant productivity (Curry, 1987; Scheu, 2003). Anthropogenic global change is presently modifying environmental factors that can impact the engineering activity of EWs (tunneling, cast production) – either indirectly via bottom-up plant responses to rising atmospheric CO_2_ (Zaller and Arnone, 1997) or by changes in plant species diversity (Zaller and Arnone, 1999b; Arnone et al., 2013) or directly via changes in amounts of precipitation (Zaller and Arnone, 1999a). Thus, a mechanistic understanding of how earthworm density (EWD, or community size) itself may influence aboveground net primary productivity (ANPP) under changing climatic conditions – especially altered amounts of growing season rainfall (IPCC, 2007) – is important.

While many greenhouse pot studies have shown that the presence of EWs can stimulate plant growth in the short term [79% of the 67 studies reviewed by Scheu (2003); with the remaining studies reporting zero or slightly negative EW effects on plant growth], only two studies have specifically quantified EW effects by experimentally manipulating their densities in field plots (Blair et al., 1997 – showing moderate stimulatory effects on ANPP; Zaller and Arnone, 1999a – showing no effects on ANPP). Many fewer studies have quantified EW effects on root growth. These studies have reported enhancement of root growth (e.g., Wurst et al., 2008; Zaller et al., 2011), reductions in root growth (cf. Scheu, 2003), or no effect on root growth (e.g., Eisenhauer et al., 2009). In cases where increased shoot growth was found, some of this increase may have resulted from a general increase in soil nutrient availability or from a stimulation of root growth into nutrient-rich EW casts (Hirth et al., 1997, 2005; Zaller and Arnone, 1999c; Decaens et al., 2001; Zaller et al., 2013). Alternatively, increased shoot growth may have resulted from shifts in plant growth (biomass) allocation toward shoots of all or some species present in plant communities in response to increases in soil nutrient availability (e.g., Lambers et al., 1998) that resulted from EW casting and soil bioturbation. However, in cases where no stimulation of shoot biomass production was observed, the extent to which EWs may have caused these effects by somehow impeding root growth (e.g., Baylis et al., 1986; Cortez and Bouché, 1992; Gunn and Cherrett, 1993; Fisk et al., 2004; Birkhofer et al., 2011) is unclear. No studies appear to have specifically quantified how EWs affects root system size (e.g., root length density, RLD) and temporal dynamics in natural plant communities. Yet, a quantitative understanding of how EWs affect root systems of native grasslands is necessary as a basis for assessing how global anthropogenic change will alter the function of these ecosystems (e.g., Zaller and Arnone, 1997, 1999c; Arnone et al., 2013).

In an earlier study (Zaller and Arnone, 1999a) in which we manipulated EWDs in field plots in a native plant species-rich calcareous grassland in NW Switzerland, we found that experimentally increasing EWDs also increased EW activity (measured in surface cast production) but did not change ANPP, even when the period of seasonal EW activity and plant growth was extended through application of artificial rains. While additional rainfall stimulated ANPP by 30% in that study (mean of 440 g m^-^^2^ year^-^^1^ in plots with natural rainfall and mean of 580 g m^-^^2^ year^-^^1^ in plots with additional rain) primarily by enhancing the growth of graminoid species (Zaller and Arnone, 1999a), the lack of effects of EWDs on ANPP in that study was surprising because previous studies in these calcareous grasslands have shown that increases in EW activity increased soil nutrient availability (Zaller and Arnone, 1997) and stimulated shoot growth (Zaller and Arnone, 1999c) and ANPP (Arnone et al., 2013). Thus, the results from our earlier study (Zaller and Arnone, 1999a) showing no EWD effects on ANPP, suggested that elevated EWDs may have adversely affected root systems in some way, while reduced EWDs may have somehow benefitted root systems.

Therefore, the objectives of the present study were to quantify the effects of EWD and rainfall treatments imposed by Zaller and Arnone (1999a) on plant community RLD to evaluate whether possible earthworm-induced changes to the root systems of these intact native grassland plant communities can mechanistically explain the lack of ANPP response reported by Zaller and Arnone (1999a).

## MATERIALS AND METHODS

Because the results presented here represent the analysis of a second data set generated during the Zaller and Arnone (1999a) study (which focused on aboveground ANPP responses to EWD and rainfall), the material and methods described in that paper apply here, as well. However, for the sake of completeness and convenience, we summarize critical elements of the methods here, and provide data from Zaller and Arnone (1999a) that describe the effectiveness of the EWD and rain treatments.

### SITE DESCRIPTION

The calcareous grassland we studied is located on a 20° southwest-facing slope near the village of Nenzlingen (canton Basel-Land), NW-Switzerland (500 m a.s.l., 47°27’ N, 7°34’ E). Mean annual precipitation is about 920 mm and mean air temperatures of about 8.5°C (Ogermann et al., 1994). Up to 1993 this grassland had been used for extensive cattle grazing and since 1993 the area has been fenced and mown twice a year in spring and autumn. Among the 100 vascular plant species found on this site, the grass *Bromus erectus* L. is dominant (Huovinen-Hufschmid and Körner, 1998). Soils are classified as a transition Rendzina (pH is about 6.5, bulk density of the top soil 1.1 g cm^-^^3^, C-to-N ratio about 12), with a well developed, stone-free, loamy topsoil and a rapid transition at 15–25 cm depth to the underlying calcareous scree material (Ogermann et al., 1994).

### EXPERIMENTAL DESIGN

The design used in our study was identical to the one described in Zaller and Arnone (1999a). To control EWD, 30 1 × 1 m plots were trenched to a depth of 45 cm with 1-mm-mesh nylon window screen in spring 1995. The screen extended 15 cm above the soil surface to create an aboveground EW barrier. Trenching to 45 cm in these shallow soils would be expected to strongly limit subsurface lateral movement of EWs into or out of the plots. Plots were arranged in a randomized complete block design (five blocks), with three EW densities (low, ambient, and high) and two amounts of rainfall (ambient and 280 mm year^-^^1^ additional rain). These amounts of added rain were applied to the appropriate plots during dry periods in the growing season to maintain suffcient soil moisture for EWs to stay active in all but the driest periods. Volumetric soil water content was continuously monitored over the topmost 10 cm of the topsoil using time-domain-reflectrometry (one measurement every 20 min). We also continuously recorded soil temperature in each plot with thermistors placed at depths of 5 and 15 cm (one reading h^-^^1^). Time courses of soil water content and soil temperature over the experimental period are presented in Zaller and Arnone (1999a).

Earthworm density treatments were established in May 1996 by first extracting EWs from each plot by applying an electrical current to moist soil (Thielemann, 1986; Ketterings et al., 1997). This non-destructive method has been shown to provide comparable estimates of EW community size and composition to other more conventional sampling methods, as long as EWs are sampled at times when they are active and when soil moisture is sufficient (Schmidt, 2001). A total of nine EW species were collected (nomenclature follows Bouché, 1972) representing three ecological groups (Bouché, 1977): anecics (*Nicodrilus longus* Ude., *N. nocturnus* Ev., *Lumbricus terrestris* L.), endogeics (*N. caliginosus* Sav., *Allolobophora chlorotica* Sav., *A. rosea* Sav., *Octolasion cyaneum* Sav.) and epigeics (*L. castaneus* Sav., *Dendrobaena mammalis* Ger.).

We created field plots with three levels of experimental EW densities (low, ambient, and high) using the following procedure. All of the EWs collected from each plot in each experimental block (six plots per block) was temporarily placed in pale containing cool water (see above). Worms in the pale were then sorted into one of three ecological groups, with worms from each ecological group placed temporarily in a smaller polyethylene beaker filled with cool water. One-sixth of the worms from each beaker were placed on the mowed surface of each of the two ambient density plots in that block. One-third of the worms from each beaker were placed on the surface of the two high density plots in that block, and no worms were placed in the plots assigned to the low EWD treatment. This procedure was repeated for each of the five blocks in May 1996 (start of density treatment) and again in September 1996 and May 1997 to maintain density treatments. All worms reentered the soil immediately after being placed on the surface of the plots. We were unable to achieve complete EW removal from low density plots because not all worms are able to exit the soil during application of electrical stimulation. Details of aboveground plant biomass sampling and harvesting procedures are described in Zaller and Arnone (1999a). The total amount of plant biomass harvested in May and September 1997 was used to estimate ANPP.

Cumulative surface cast production (dry mass) was measured biweekly during periods of highest EW activity (Zaller and Arnone, 1997) from October 1996 to May 1997 on one of the two 25 × 25 cm sub-plots in each plot as an indicator of EW activity. After weighing cast fresh mass in the field on a portable balance, it was returned to its original position and deformed slightly to facilitate the identification of newly produced casts at the next sampling date. Cast subsamples from each plot were taken at each sampling date to calculate fresh mass-to-dry mass ratios (80°C, 24 h).

Results published Zaller and Arnone (1999a) showed that our manipulation of EW community size was effective, although EW populations fluctuated between sampling dates with community size (number and biomass) tending to increase slightly in low density plots, tending to decrease slightly in high density plots, but remaining largely unchanged in ambient density plots. Averaged across the experimental year, Zaller and Arnone (1999a) found that low density plots contained the fewest EWs (37 ± 5 worms m^-^^2^) and least biomass (26.7 ± 3.3 g m^-^^2^). The ambient density plots contained about twice the number and biomass of the low density plots. The high density plots contained about 50% more worms and 50% more biomass than the ambient plots contained. Zaller and Arnone (1999a) further demonstrated that increasing EWD also resulted in significant increases in EW activity measured as cumulative surface cast production). However, additional rain had no detectable effect on the size of EW communities in any EWD treatment even though soil water content was consistently greater in these plots compared to plots receiving no additional water (Zaller and Arnone, 1999a). Daily mean soil temperature in all plots fluctuated in a normal fashion with season, but did not differ significantly among worm density or rain treatments, or between 5 and 15-cm soil depths, at any time during the study (Zaller and Arnone, 1999a).

### MEASURING ROOT LENGTH DENSITY USING MINIRHIZOTRONS

In late April 1995 we installed one transparent minirhizotron tube (5 cm in diameter, 100 cm long) in each experimental plot at an angle of about 35° to the plane of the soil surface. The tubes were inserted through the A horizon and into the upper 3 cm of the rocky subsoil allowing us to observe roots in the top 18 cm of soil, the layer in which 80% of all roots occur (Arnone et al., 2000). Before installing the tubes we etched an observation track (18 mm wide, 54 cm long) on the outside upper surface of each tube. We then divided each track into 45 frames, each 12 mm high and 18 mm wide. The tube bottoms were capped before insertion into pre-cored cylindrical holes, and the top 10 cm of each tube wrapped in opaque tape and stoppered to prevent light penetration and entry of debris and insects.

We were unable to distinguish among roots of the more than 30 species growing in each experimental plot and thus only considered responses of the root system of the entire plant community. In April 1996, we recorded video images of roots in all 45 frames in each minirhizotron using a Bartz BCT-2 Minirhizotron Camera (Bartz Technology Co., Santa Barbara, CA, USA) attached to a Hi-8 Sony Camcorder (all mounted on a backpack). We repeated this on 11 more dates up to April 1997. All 45 frames along the tubes were used to quantify (RLD, cm root cm^-^^2^ minirhizotron tube surfaces) through the soil profile. This was accomplished by viewing undigitized video tapes and counting intersects with gridlines drawn on an overhead transparency and placed over the video monitor (Tennant, 1975). Average RLD during the first year (April 1996–April 1997) was calculated for each minirhizotron observation frame using the Tennant (1975) method and expressed as cm of root length per cm^2^ of minirhizotron observation area (Smit et al., 2000).

### CALCULATIONS AND STATISTICAL ANALYSIS

In all analyses we used the plot as the experimental unit. First, we tested the effects of EWD, additional rain, sampling date, and their interactions, on RLD using a three-way analysis of variance (ANOVA) model. In these ANOVAs, the EWD effect was tested against the EWD × Block term, the Rain effect was tested against the Rain × Block term, the Sampling date effect was tested against the residual term, and the EWD × Rain effect was tested against the EWD × Rain × Block term. We also used two-way ANOVAs to explore EWD effects within each Rain treatment to further elucidate the possible occurrence of significant (*P* < 0.05) EWD × Rain interactions. In these ANOVAs, the EWD effect was tested against the EWD × Block term, and the Sampling date effect was tested against the residual term, and the EWD × Sampling date effect was tested against the EWD × Sampling date × Block term. Because the block effect was never statistically significant (*P* > 0.05), this factor was removed from all ANOVAs. Second, the effect of additional rain on RLD over time was tested using repeated measures ANOVA (von Ende, 1993) for each soil depth, and the sum of all depths, and EWD. Third, we used Pearson correlations (e.g., Zar, 1998) to test *a priori* linear relationships between RLD and EW activity (cast production), RLD and EWD, RLD and EW biomass, and RLD and annual net aboveground (shoot) plant biomass production. We used a 3-parameter asymptotic regression fitting procedure for non-linear exponential relationships (StataCorp, College Station, TX, USA). Data were transformed before ANOVA as necessary to ensure homogeneity of variance and normal distributions. All statistical analyses were performed using Stata version 11.1. Values given throughout the manuscript are means ± SEs.

## RESULTS

**FIGURE 1:**
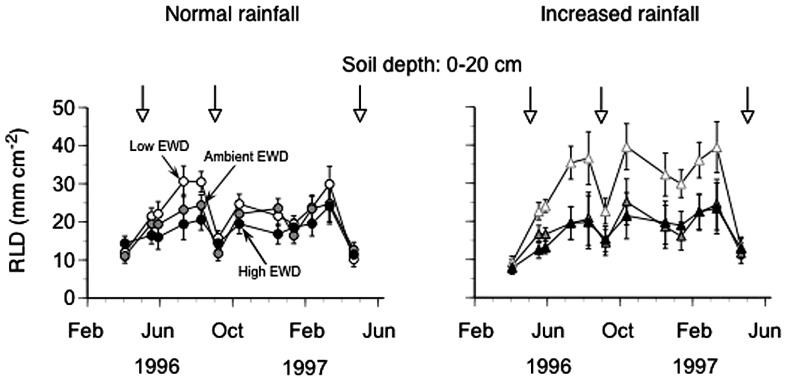
**Seasonal dynamics of root length density (RLD) measured using minirhizotrons (0–20 cm topsoil, one 60 cm long tube per plot) in trenched grassland plots with manipulated earthworm densities (EWDs) under ambient and increased rainfall (means ± SEs, *n* = 5 plots per experimental treatment; treatment effects analyzed using repeated measures ANOVAs – see Table 1).** Open symbols represent low EWD; gray symbols: ambient EWD; and black symbols: high EWD. Circles indicate ambient rainfall means, and triangles increased rainfall means.

Root length densities averaged across all depths (0–20 cm) increased during the growing season starting in April reaching peak values in August 1995 in all plot regardless of EWD treatment (**Figure 1**; **Table 1**). These maximum RLDs were maintained throughout the cold season into mid-March 1996, at which point RLDs in all EWD treatments decreased by mid-May 1996 to densities measured in May of the previous year that in all EWD treatments corresponded to RLDs measured in plots kept at natural EW densities. This temporal pattern was discernable at all of the depths in the upper soil layer (data not shown).

**Table 1:** Results of analyses of variance (ANOVA) quantifying treatment effects of earthworm density (EWD) and additional simulated rainfall (Rain) on root length density (RLD) measured in the topsoil (0–20 cm) of 1 × 1 m experimental plots in native calcareous grassland in the Jura hills of northwestern Switzerland.

Reference figure	Factor	F-value	df	*P*-value	Note
1	EWD	5.06	2,12	**0.0254**	All data
	Rain	0.41	1,4	0.5569	
	Date	2.75	11,319	0.0809	
	EWD × Rain	1.69	2,8	0.2446	
1	EWD	0.80	2,12	0.4727	Natural rain
	Date	16.44	11,132	**<0.0001**	only
	EWD × Date	1.34	22,132	0.1640	
1	EWD	5.65	2,12	**0.0187**	Added rain
	Date	27.22	11,132	**<0.0001**	only
	EWD × Date	2.43	22,132	**0.0010**	
2	EWD	5.06	2,12	**0.0254**	All data
	Rain [R]	0.41	1,4	0.5569	
	Soil depth [D]	4.31	2,8	**0.0437**	
	EWD × Rain	1.69	2,8	0.2446	
	EWD × Depth	0.66	6,36	0.6801	
	Rain × Depth	3.92	3,12	**0.0365**	
	EWD × R × D	0.28	6,24	0.9392	

**FIGURE 2:**
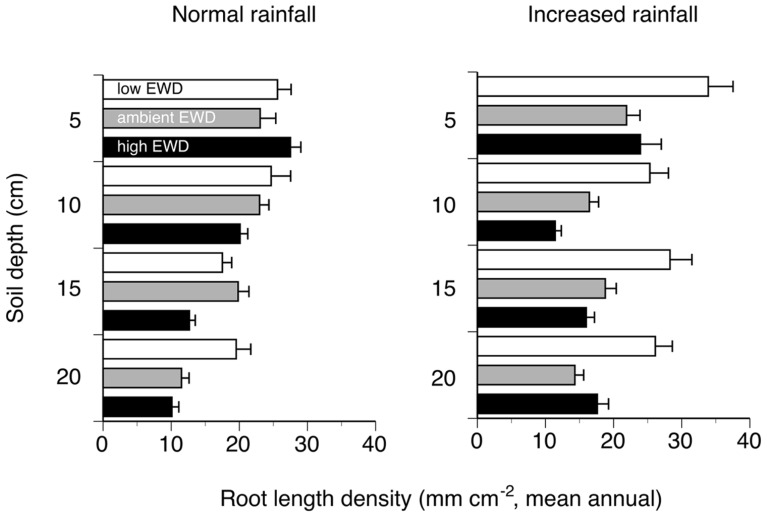
**Mean annual root length density in different soil depths measured using minirhizotrons (0–20 cm topsoil, one 60 cm long tube per plot) in trenched grassland plots with manipulated earthworm densities (EWDs) under ambient and increased rainfall (means ± SEs, *n* = 5 plots per experimental treatment; treatment effects analyzed using a three-way ANOVA – see Table 1)**.

*Repeated measure ANOVA was used to analyze treatment effects in the seasonal time courses of RLD (Reference Figure 1). A three-way ANOVA was used to analyze treatment effects on RLD at different depths in the topsoil (Reference Figure 2).*

Overall, reducing EWD below natural levels increased RLDs when viewed across all depths, while increasing EWD above natural levels had no detectable effects (**Figure 1**; **Table 1**). These patterns were also apparent when comparing mean annual RLDs measured at different soil depths (**Figure 2**; **Table 1**). However, the statistically significant EWD effects detected in ANOVAs were primarily due to the stimulation of RLDs in low EWD plots that received additional simulated rain (**Table 1**).

**FIGURE 3:**
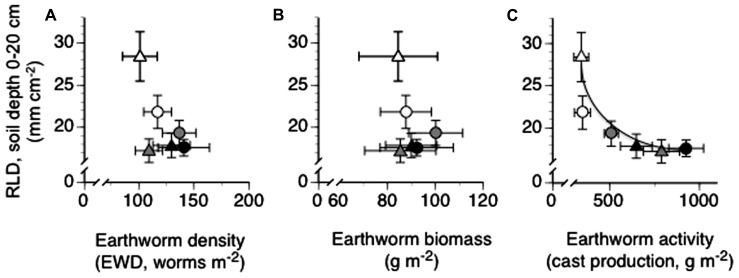
**Relationships between mean annual root length density (RLD) and mean annual earthworm density (A), mean annual earthworm biomass (B) and mean cumulative annual earthworm activity – cast production (C) in trenched grassland plots with manipulated earthworm densities (EWDs) under ambient and increased rainfall (means ± SEs, *n* = 5 plots per experimental treatment) analyzed using both simple linear regression and exponential best fit algorithms (see Materials and Methods) of mean treatment values**. Scatter plots with no best fit lines shown indicate a lack of a statistically significant (*P* < 0.05) slope or curve. Symbology for treatment given in legend to **Figure 1**.

Mean annual RLD viewed across all depths and treatment combinations appeared to be unrelated to mean annual EWD or mean annual EW biomass (**Figure 3A**: *P*_slope_ = 0.1566, **Figure 3B**: *P*_slope_ = 0.4290). However, mean annual RLD was highly related to mean annual surface cast production, with RLD decreasing exponentially with increasing EW surface cast production (**Figure 3C**: *P*_slope_ = 0.0055, *r*^2^ = 0.88).

**FIGURE 4:**
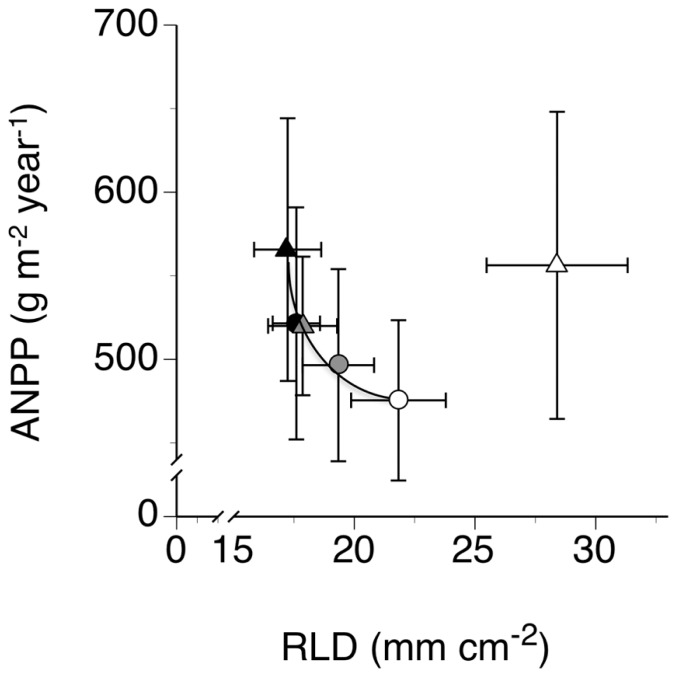
**Relationships between mean annual aboveground plant biomass production (ANPP) and mean annual root length density (RLD) in trenched grassland plots with manipulated earthworm densities (EWDs) under ambient and increased rainfall (means ± SEs, *n* = 5 plots per experimental treatment) analyzed using both simple linear regression and exponential best fit algorithms (see Materials and Methods) of mean treatment values but excluding the treatment mean for the low EWD and increased rainfall (upper rightmost point).** Symbology for treatments given in the legend to **Figure 1**.

The scatter diagram (**Figure 4**) of treatment mean annual ANPP measured during the study year plotted on corresponding treatment mean annual RLD, calculated across all depths, indicated a lack of significant relationship between ANPP and RLD. However, when we removed from the scatter diagram the point in the upper right (outlier), we observed a significant negative exponential relationship between the two variables, with ANPP declining precipitously with increasing RLD (*P* = 0.0070, *r*^2^ = 0.94; **Figure 4**). This point represented the treatment mean from the low EWD plots that received additional rain.

## DISCUSSION

This study for the first time shows that during the course of 1 year, EW densities significantly affected the size (length density) of native grassland root systems, particularly when EWDs were kept low and when sufficient soil moisture was present (**Figure 1**).

Increased RLDs observed under low EWDs (especially under increased rainfall), relative to RLDs measured under ambient and high EWDs, may have occurred for a number of possible reasons. These possibilities include: (i) an increased need for plants in these communities to invest in root production (e.g., plant functional equilibrium adjustment; e.g., Lambers et al., 1998) to forage for lower levels of available nutrients (Fitter, 1994; Hutchings et al., 2000) relative to plants in ambient and high EWD plots as EWs increase soil nutrient availability (Zaller and Arnone, 1997; Arnone et al., 2013); (ii) lower root nutrient uptake efficiency as suggested by results of Fisk et al. (2004) with more plant carbon invested in root tissue growth per unit of nutrient taken up; (iii) reduced physical disturbance by EWs of newly formed root tips (which has not yet been experimentally addressed); or (iv) a reduction in possible root herbivory under lower EWDs (suggestive indirect evidence: Baylis et al., 1986; Gunn and Cherrett, 1993; Birkhofer et al., 2011).

The lack of an increase in RLDs under ambient and high EWDs (observed under both ambient and increased rainfall), relative to RLDs measured under low EWDs, also may have occurred for a number of reasons. These possibilities include: (i) a reduced need for plants to invest in root production where EWs increased soil nutrient availability; (ii) greater root nutrient uptake efficiency; (iii) increased physical disturbance by EWs of newly formed root tips; or (iv) an increase in possible root herbivory. Potential facilitation of root growth in high EWD plots through enhanced creation of EW channels observed in other studies (Springett and Gray, 1997; Pitkänen and Nuutinen, 1997) did not seem to be operative in our ecosystems where we actively manipulated EWDs [as Carpenter (1985) also found].

Our finding that RLD was substantially lower during the vegetation period (growing season) than during winter, when EWs were less active, suggests a possible wintertime reduction in “negative” EW-induced effects on RLD. Observed temporal fluctuations in RLDs (**Figure 1**) in all treatments indicate that neither EWD treatments nor rainfall additions altered normal seasonal behavior of root systems of these native plant communities. Not surprisingly, supplemental rain caused deeper infiltration of water into soils of these plots than occurred in plots receiving only ambient rain. Higher moisture at depth promoted root growth that resulted in higher RLDs at depth in plots receiving additional rain.

The presence of a significant negative relationship between RLD and EW activity (**Figure 3C**), and the absence of significant relationships between RLD and EWD (**Figure 3A**) or EW biomass (**Figure 3B**), indicate that EW bioturbation may be primarily responsible for reductions in RLD under high EWDs (**Figure 1**). However, the mechanism(s) by which increases (high EWDs) and decreases (low EWDs) in bioturbation may have acted to reduce (high EWDs) or enhance (low EWDs), respectively, RLDs is unclear.

Physical disruption of root growth through bioturbation under high EWDs, and release from disruption under low EWDs, could explain the patterns we observed in RLD. However, according to the principles of shoot:root functional equilibrium (e.g., Brouwer, 1963; Thornley, 1972; Iwasa and Roughgarden, 1984; Lambers et al., 1998), if RLDs were reduced under high EWD because of physical damage to roots, then growth (biomass) should be allocated to roots away from shoots leading to lower ANPP and higher root mass fractions (RMFs). However, our data do not show that this occurred.

A more likely explanation of bioturbation effects supported by our results (**Figure 4**) involves functional equilibrium growth shifts in response to changes in soil nutrient mineralization and soil nutrient availability (cf., Lee, 1985; Edwards and Bohlen, 1996; Willems et al., 1996; Zaller and Arnone, 1997; Görres et al., 2001; Whalen et al., 2001; Araujo et al., 2004). These results showed (a) no change in ANPP (Zaller and Arnone, 1999a) but reductions in RLDs under high EWDs, and (b) no change in ANPP and increases in RLDs under low EWDs (**Figure 1**). Thus, our data indicate that the following two treatment response paths likely occurred in our study. (1) High EWDs led to high bioturbation, high microbial and EW (via casting) nutrient mineralization, high plant nutrient availability, low RLDs (reduced need for plants to invest in nutrient foraging organs, – e.g., Fitter, 1994; Hutchings et al., 2000), and low RMFs. (2) Low EWDs led to low bioturbation, low microbial, and EW nutrient mineralization, low plant nutrient availability, high RLDs (increased need for plants to invest in nutrient foraging organs), and high RMFs (**Figure 4**).

Together, the results of our study conclusively show that increasing EW activity can reduce the size of native grassland root systems in the field that, in the short term, do not appear to affect ANPP. In the longer term, however, it is unclear whether (a) bioturbation from large EW populations could lead to greater nutrient leaching from soils (Bohlen et al., 2004) that lead to reductions of ANPP; or (b) the absence of any increase in ANPP in grassland ecosystems under high EW populations (Zaller and Arnone, 1999a) would continue to provide sufficient carbon inputs to support such large EW populations. Finally, our results suggest that EWDs may mainly affect RLDs and plant community aboveground vs. belowground growth (biomass) allocation under climate changes that include more frequent wetter-than-average growing seasons.

## AUTHOR CONTRIBUTIONS

Both authors jointly developed the concept of this experiment, conducted the measurements, analysed the data, and wrote the manuscript.

## Conflict of Interest Statement

The authors declare that the research was conducted in the absence of any commercial or financial relationships that could be construed as a potential conflict of interest.
